# Hyperspectral Imaging and Machine Learning for Diagnosing Rice Bacterial Blight Symptoms Caused by *Xanthomonas oryzae* pv. *oryzae*, *Pantoea ananatis* and *Enterobacter asburiae*

**DOI:** 10.3390/plants14050733

**Published:** 2025-02-27

**Authors:** Meng Zhang, Shuqi Tang, Chenjie Lin, Zichao Lin, Liping Zhang, Wei Dong, Nan Zhong

**Affiliations:** 1 College of Engineering, South China Agricultural University, Guangzhou 510642, China; zhangmeng@aaas.org.cn (M.Z.);; 2 Agricultural Economy and Information Research Institute, Anhui Academy of Agricultural Sciences, Hefei 230031, China; 3 Guangdong Provincial Key Laboratory of Agricultural Artificial Intelligence (GDKL-AAI), Guangzhou 510642, China; 4 National Center for International Collaboration Research on Precision Agricultural Aviation Pesticide Spraying Technology, Guangzhou 510642, China

**Keywords:** *Pantoea ananatis*, *Enterobacter asburiae*, *Xanthomonas oryzae* pv. *oryzae*, rice bacterial blight, hyperspectral imaging, convolutional neural networks, generative adversarial networks

## Abstract

In rice, infections caused by *Pantoea ananatis* or *Enterobacter asburiae* closely resemble the bacterial blight induced by *Xanthomonas oryzae* pv. *oryzae*, yet they differ in drug resistance and management strategies. This study explores the potential of combining hyperspectral imaging (HSI) with machine learning for the rapid and accurate detection of rice bacterial blight symptoms caused by various pathogens. One-dimensional convolutional neural networks (1DCNNs) were employed to construct a classification model, integrating various spectral preprocessing techniques and feature selection algorithms for comparison. To enhance model robustness and mitigate overfitting due to limited spectral samples, generative adversarial networks (GANs) were utilized to augment the dataset. The results indicated that the 1DCNN model, after feature selection using uninformative variable elimination (UVE), achieved an accuracy of 86.11% and an F1 score of 0.8625 on the five-class dataset. However, the dominance of *Pantoea ananatis* in mixed bacterial samples negatively impacted classification performance. After removing mixed-infection samples, the model attained an accuracy of 97.06% and an F1 score of 0.9703 on the four-class dataset, demonstrating high classification accuracy across different pathogen-induced infections. Key spectral bands were identified at 420–490 nm, 610–670 nm, 780–850 nm, and 910–940 nm, facilitating pathogen differentiation. This study presents a precise, non-destructive approach to plant disease detection, offering valuable insights into disease prevention and management in precision agriculture.

## 1. Introduction

**Figure 1:**
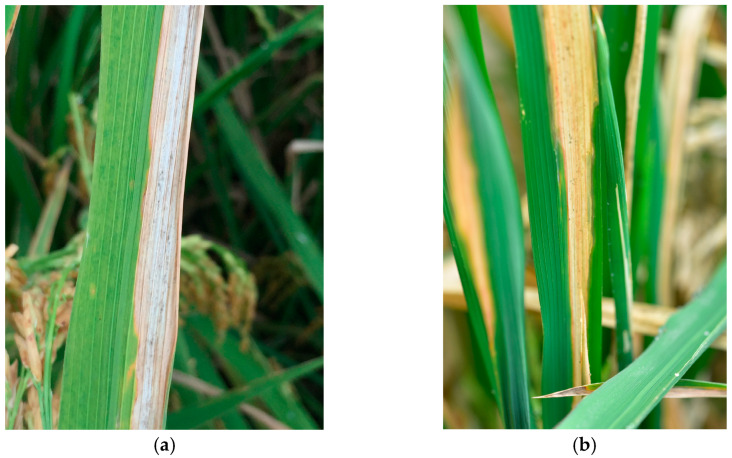
Comparison of rice bacterial blight with similar symptoms caused by different bacterial strains in field conditions: (**a**) *Xanthomonas oryzae* pv. *oryzae* and (**b**) *P. ananatis* and *E. asburiae*.

Rice is a staple crop that sustains more than half of the global population and is primarily cultivated in the monsoon regions of Asia. Rice production encounters various biological challenges [1]; among these, rice bacterial blight caused by *Xanthomonas oryzae* pv. *oryzae* (*Xoo*), is one of the most devastating. The pathogen enters rice plants through wounds or stomata, colonizes vascular bundles, and proliferates rapidly. As the infection progresses, the pathogen spreads throughout the plant, causing severe tissue damage and significant yield losses [2]. A newly emerging rice disease caused by *Pantoea ananatis* (*P. ananatis*) and *Enterobacter asburiae* (*E. asburiae*) exhibits symptoms similar to bacterial blight, making visual differentiation between the two diseases challenging. This disease spreads rapidly, exhibits high pathogenicity, and significantly reduces rice yields. For effective management, accurate identification and differentiation of these diseases are essential. Representative symptoms of the new disease are illustrated in Figure 1.

Rice diseases caused by *P. ananatis* or *E. asburiae* have been documented in various countries and regions. The first report of rice bacterial blight induced by *P. ananatis* was documented in India in 2008 [3]; subsequent cases were reported in West African countries such as Benin and Togo between 2011 and 2015 [4]. In recent years, similar outbreaks have been reported in Russia [5], Malaysia [6], the United States [7] and various regions in China. Between 2016 and 2017, rice samples exhibiting symptoms resembling bacterial blight were identified in Dandong (Liaoning), Gongzhuling, and Laxi (Jilin), where *P. ananatis* was confirmed as the pathogen [8]. By 2020, similar cases in the Zhejiang and Jiangxi provinces were also attributed to *P. ananatis* [9,10]. In July 2021, rice plants exhibiting symptoms akin to bacterial leaf streaks were identified near Linyi City, Shandong Province, and were attributed to *P. ananatis* [11]. Furthermore, analyses by Xue et al. [12] of rice samples collected from Sichuan (2017) and Guangdong (2020) identified both *P. ananatis* and *E. asburiae* as the pathogens responsible for bacterial blight symptoms. These findings underscore the widespread emergence of *P. ananatis* and *E. asburiae* as the primary pathogen in rice-growing regions. Notably, its transmission modes [13], pathogenic mechanisms [14], and treatment resistance [15] differ significantly from those of bacterial blight caused by *Xoo*. Conventional plant disease diagnosis is predominantly dependent on field expertise or reagent-based assays, which require specialized knowledge and are constrained by high costs and limited scalability. Xu et al. [16] utilized the *acnA* gene of *P. ananatis* to design 22 specific primer pairs via sequence analysis, integrating these with existing primers for *Xoo*. This led to the development of a duplex polymerase chain reaction (PCR) assay that accurately detected both pathogens in rice samples. Similarly, Wang et al. [17] developed a rapid and precise detection method for *P. ananatis* employing a recombinase-aided amplification detection system combined with a lateral flow dipstick, which targeted the *GyrB* gene. This system enables early pathogen detection without the need for DNA extraction or specialized equipment. While these methods effectively identify pathogenic bacteria, their reliance on reagent kits increases costs and generates waste, thereby limiting their practicality for large-scale testing. Therefore, the development of rapid, accurate, and non-destructive diagnostic methods is essential to fulfill the requirements of large-scale agricultural applications.

Hyperspectral imaging (HSI) is an advanced, non-destructive technique for crop disease detection, exploiting the unique spectral absorption patterns of atomic and molecular structures to infer the physical and chemical composition of plant tissues [18,19]. This technology enables both qualitative and quantitative analyses of key components [20]. Pathogen infection induces significant alterations in the internal chemical markers of crops. Unlike conventional diagnostic approaches that predominantly assess visible lesion morphology, HSI exploits crop reflectance properties for disease detection [21,22]. This capability enables the detection of both internal and external structural alterations, as well as changes in biochemical composition, thereby providing insights into the extent of pathogen invasion. Supported by advances in statistical and machine learning techniques, HSI has become an indispensable tool for plant disease detection, offering superior sensitivity compared to traditional visual inspection or reagent-based methods. In this study, we aimed to evaluate and compare the performance of several machine learning algorithms for plant disease detection using HSI data. The primary focus was on testing the effectiveness of traditional machine learning models, such as partial least squares discriminant analysis (PLSDA), k-nearest neighbors (KNNs), and random forest (RF), alongside deep learning models, specifically one-dimensional convolutional neural networks (1DCNNs). The models were trained and evaluated on hyperspectral data collected from crops affected by different pathogens. Hyperparameter tuning was performed for each model to optimize performance, and the models were evaluated using a testing set to assess their generalization capabilities. Ugarte Fajardo et al. [23] applied PLSDA to hyperspectral data to detect black Sigatoka, identifying critical wavelength ranges of 577–651 nm and 700–1019 nm, and achieved a prediction accuracy of 98%. Lu et al. [24] employed principal component analysis (PCA) on hyperspectral data from tomato leaves affected by various diseases, combining it with KNN to classify the top 30 spectral indices. This method achieved 100% accuracy for healthy leaves, while the classification accuracy for infected leaves improved with increasing disease severity. Zhang et al. [25] developed RF models to detect wheat fusarium head blight at various growth stages by selecting characteristic bands for each stage, achieving root mean square errors below 0.08. The integration of machine learning with hyperspectral data has further advanced through the application of deep learning techniques. Feng et al. [26] employed 1DCNN to classify rice blast severity, achieving an overall accuracy of 98.58%. In another study, Feng et al. [27] combined hyperspectral data with a 1DCNN to classify bacterial infections in four rice seedling varieties inoculated with three bacterial solutions, attaining over 88% accuracy in the testing set. Collectively, these studies underscore the significant potential of HSI, particularly when integrated with machine learning, for the accurate and efficient detection of plant disease.

Although 1DCNN surpasses traditional machine learning approaches in feature extraction and generalization, hyperspectral data acquisition remains expensive and time-intensive, while deep learning necessitates extensive training datasets for optimal performance. These factors create significant challenges for agricultural applications, where data acquisition can be a major barrier [28,29]. Furthermore, although HSI can capture a wealth of information related to plant diseases, the high-dimensional nature of the data poses challenges for both computational efficiency and model generalizability. Notably, for rice bacterial blight caused by *P. ananatis* and *E. asburiae*, no research has yet demonstrated the feasibility of using HSI for classification. Thus, systematically correlating spectral data with disease characteristics is crucial for identifying unique spectral signatures and constructing robust diagnostic models.

To address the challenges in identifying rice bacterial blight caused by various pathogens, this study focuses on three main aspects: (1) constructing a high-throughput dataset of rice bacterial blight symptoms caused by various pathogens using HSI and generative adversarial networks (GANs) to meet modeling requirements; (2) conducting a comparative analysis that integrates disease morphological characteristics, spectral curves, and PCA to elucidate the similarities and differences in the infection process and feature variations in rice leaves caused by various pathogens; and (3) comparing multiple preprocessing, feature selection, and modeling algorithms to develop an efficient and accurate 1DCNN model based on spectral reflectance for classifying rice bacterial blight symptoms caused by various pathogens, while identifying the key spectral bands for distinguishing these lesions.

## 2. Results

### 2.1. Spectral Characteristics

**Figure 2:**
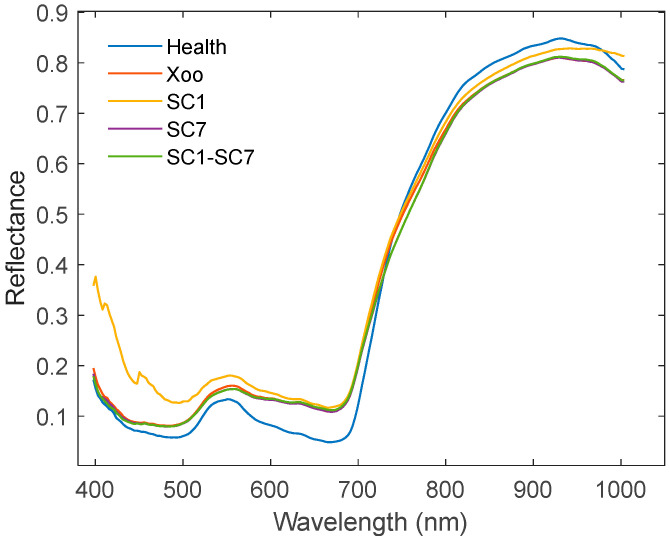
Spectral profiles of healthy rice leaves and rice leaves infected with different bacteria. Health represents healthy rice leaves, Xoo represents leaves inoculated with pathogen *Xoo* PXO99A, and SC1, SC7, and SC1–SC7 represent leaves inoculated with the *E. asburiae* strain SC1, the *P. ananatis* strain SC7, and their mixed pathogen, respectively.

Figure 2 presents the average spectra of five groups of rice leaves in the 400–1000 nm wavelength range. Although distinct differences in reflectance are observed among healthy, SC1, and the other sample groups in specific wavelength ranges, the overall spectral curves of all five groups exhibit similar trends. Notably, the spectral curves of Xoo, SC7, and SC1–SC7 overlap almost completely, suggesting that pathogen infection alters only a limited subset of leaf components, thereby making differentiation among the groups challenging. In the visible spectrum (400–720 nm), infected leaves exhibit higher reflectance than healthy leaves, especially in the 450–500 nm and 560–680 nm range, indicating reduced chlorophyll absorption as a result of pathogen stress. SC1 shows the most pronounced increase in reflectance within this range, likely due to pathogen-induced pigment alteration. A distinct peak is observed between 520 and 570 nm, while valleys occur at 440–500 nm and 660–680 nm, emphasizing the significance of chlorophyll absorption bands in disease differentiation. In the near-infrared region (780–1000 nm), healthy leaves display the highest reflectance, followed by SC1, whereas Xoo, SC7, and SC1–SC7 exhibit lower reflectance, potentially reflecting structural damage and reduced cellular integrity due to infection. These spectral patterns provide valuable insights into the interplay between pathogen infection and leaf biophysical properties, thereby forming the basis for subsequent feature extraction and model development.

### 2.2. PCA Explanatory

**Figure 3:**
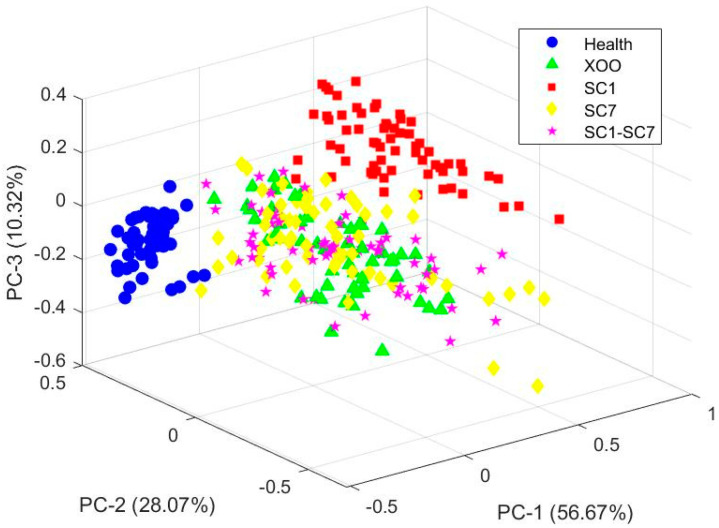
PCA result of five sample groups: Health, Xoo, SC1, SC7, and SC1–SC7.

PCA was employed to reduce the dimensionality of the 224 spectral bands, enabling three-dimensional visualization of the sample distribution among groups. In this representation, samples that are positioned closer together exhibit greater spectral similarity. As shown in Figure 3, the first three principal components cumulatively account for 95.06% of the total variance (56.67% + 28.07% + 10.32%), indicating that they capture the majority of variation in the original dataset. The PCA plot demonstrates that both the Health and SC1 groups are clearly distinguishable from the other three groups, reflecting distinct spectral features unique to these categories. Notably, the Health group forms a compact and isolated cluster, underscoring its significant divergence from the infected samples. In contrast, substantial overlap is observed among the Xoo, SC7, and SC1–SC7 groups, reflecting similar spectral characteristics. However, the SC1 group clusters along the PC1 axis suggest that its spectral features are sufficiently unique for accurate classification in contrast to the considerable overlap observed among the Xoo, SC7, and SC1–SC7 groups. The clustering patterns reveal that, while PCA effectively separates healthy samples from infected samples, advanced classification techniques, such as machine learning, are necessary to resolve the ambiguity among overlapping disease categories.

### 2.3. Data Augmentation and Evaluation

Given the limited number of spectral samples, GAN was employed to generate synthetic spectra, thereby augmenting the training dataset and mitigating overfitting. Figure S1 illustrates the progression of the generated SC7 spectrum over different training epochs, highlighting its increasing alignment with real spectra as the training iterations advanced. At 100 epochs, the generated spectrum exhibited considerable randomness and lacked discernible patterns. By 400 epochs, the spectrum began to approximate the real spectral shape, although noticeable noise persisted. As training progressed to 800 and 1200 epochs, the noise gradually diminished, and the generated spectrum increasingly resembled the actual data. By 2000 epochs, the generated spectrum closely mirrored the real spectrum, exhibiting only slight intensity differences along the vertical axis, thereby confirming the success of data augmentation. At this point, the GAN model was deemed fully trained. For each category, including SC7, 100 synthetic spectra were generated to balance the dataset and enhance model generalization.

**Figure 4:**
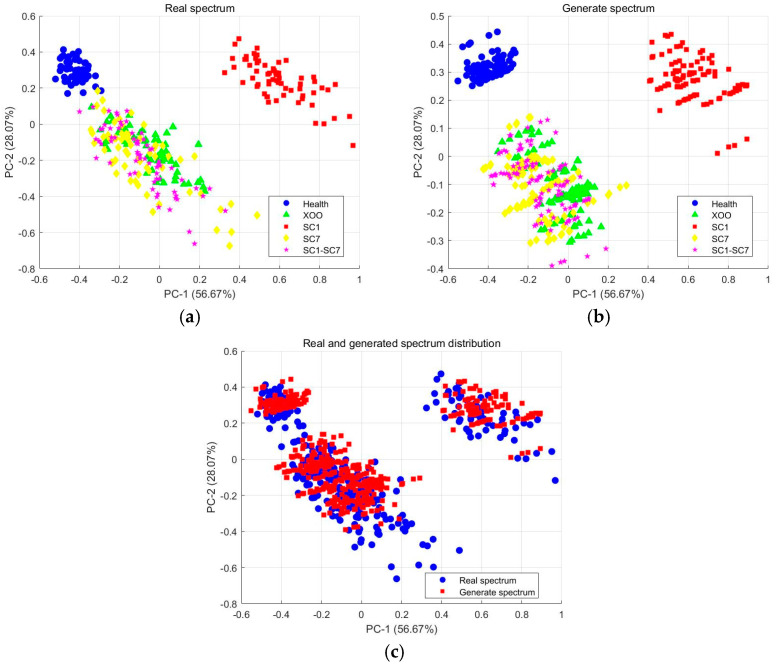
PCA results of real and generated spectra: (**a**) real spectra, (**b**) generated spectra, and (**c**) comparison of distribution between real and generated spectra.

To further evaluate the reliability of the generated spectra, a statistical analysis was conducted using PCA [30,31]. PCA reduced the dimensionality of the spectral data, thereby enabling the visualization of the distribution patterns of both real and generated spectra. As shown in Figure 4a,b, the generated spectra closely align with the distribution of real spectra, particularly in regions with a high sample density, thereby validating the effectiveness of GAN-generated data for dataset augmentation. Further analysis in Figure 4c reveals that the distribution of real and generated spectra is consistent with a significant overlap, further demonstrating their similarity. Notably, the generated spectra exhibit a higher clustering density in regions where real spectra are abundant, suggesting a strong similarity between the pseudo-spectral samples and real data, albeit with minor variations. These results confirm the high quality of the generated spectra and their suitability for augmenting the existing dataset.

### 2.4. Adulteration Detection Based on Full Spectra

To evaluate the performance of the 1DCNN model, a benchmarking analysis was conducted against three traditional machine learning algorithms: PLSDA, KNN, and RF. Each classification model underwent comprehensive parameter optimization based on expert knowledge and grid search. For the KNN model, the primary parameter tuned was the number of neighbors (k) selected to balance generalization and overfitting. In the RF model, the number of trees (n) and maximum depth (depth) were optimized to prevent overfitting and ensure stability. For PLSDA, the number of principal components (PCs) was adjusted to retain relevant information while minimizing noise. Additionally, multiple preprocessing techniques, such as the Savitzky–Golay filter (SG), normalization (NOR), baseline correction (BASE), standard normal variate (SNV), and multiplicative scatter correction (MSC), were examined to determine the most effective combinations of classification models and preprocessing methods. Both raw and preprocessed datasets were evaluated, with training and testing set accuracies serving as key performance metrics. The performance results for each model are presented in Table S1.

Traditional machine learning models, including PLSDA, KNN, and RF, demonstrated improved performance when combined with preprocessing techniques such as MSC and SG, underscoring the critical role of preprocessing in feature extraction and noise reduction. Among the various combinations, MSC-PLSDA and SG-KNN achieved the highest testing set accuracy at 81.02%, indicating the effectiveness of these preprocessing methods in enhancing model performance. For the RF model, SG preprocessing yielded the best testing set accuracy of 79.17%, further emphasizing the importance of appropriate preprocessing in improving spectral data classification. The 1DCNN model consistently outperformed traditional methods, achieving a peak testing set accuracy of 88.89% while effectively learning from raw spectral data, thereby bypassing the need for preprocessing pipelines. This underscores the capacity of deep learning models to process raw spectral data directly without complex preprocessing steps. Notably, while traditional models benefit significantly from preprocessing, the 1DCNN model maintains high performance across different preprocessing conditions, exhibiting minimal variance between raw and preprocessed datasets.

### 2.5. Identification of Important Wavelengths

**Figure 5:**
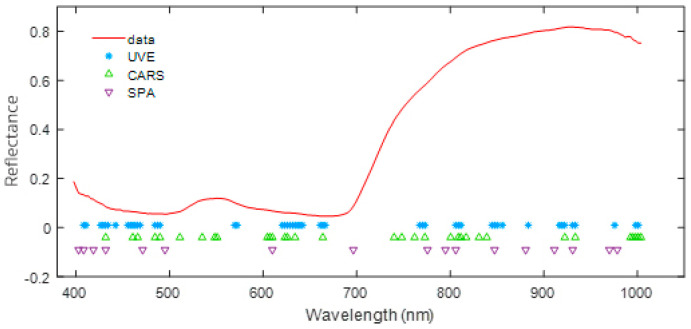
Feature band positions selected by various feature extraction methods.

Modeling with a full spectrum of data consisting of 224 wavelength points introduced excessive complexity due to high collinearity and redundant information, which increased computational demands and hindered model performance. To address these issues, we applied feature selection methods, namely uninformative variable elimination (UVE), competitive adaptive reweighted sampling (CARS), and successive projections algorithm (SPA), to identify key wavelength bands while preserving critical spectral information. These methods effectively reduced data dimensionality and computational complexity. UVE, CARS, and SPA identified 49, 33, and 17 key wavelengths, respectively. By focusing on these selected bands, model complexity, and computational demands were significantly reduced without compromising essential spectral information. The specific wavelength positions identified by each method are shown in Figure 5.

The spectra of key bands identified through feature selection methods were used for subsequent modeling and classification. The performance results for each model are summarized in Table 1. Compared to Table S1, all models experienced varying degrees of accuracy reduction after dimensionality reduction. Among them, the MSC-PLSDA, SG-KNN, SG-RF, and 1DCNN models showed the smallest declines when paired with the UVE, SPA, SPA, and UVE methods, respectively. The recognition accuracy decreased by 2.78% for MSC-PLSDA, SG-RF, and 1DCNN, and by 1.85% for SG-KNN. Despite these reductions, 1DCNN maintained the highest recognition performance, showcasing its robustness and ability to adapt effectively to reduced data complexity.

Additionally, Table 2 presents the precision, recall, and F1 scores for each data category. The Health and SC1 groups achieved near-perfect scores in the models with the best performance, highlighting their distinct and easily identifiable spectral characteristics. For the remaining three categories, the 1DCNN model consistently achieved the highest F1 scores, emphasizing its effectiveness in distinguishing complex or overlapping data categories.

Figure S2 illustrates the prediction outcomes for each data category using various modeling algorithms, presented as confusion matrices. The Health and SC1 groups achieved relatively high classification accuracy, indicating that these categories were well separated and easily distinguished by the models. However, more frequent misclassifications were observed between the Xoo and SC7 groups, with SC7 often being misclassified as Xoo. This overlap suggests that the spectral features of Xoo and SC7 are similar, leading to some ambiguity in distinguishing between these two classes. Additionally, SC7 showed a high rate of confusion with SC1–SC7, indicating some inherent similarity in the spectral characteristics of these groups. Overall, the performance across the models demonstrates that more complex algorithms, such as 1DCNN, are more capable of handling these ambiguities and distinguishing between challenging categories compared to simpler models like PLSDA, KNN, and RF. These results emphasize the importance of choosing the right modeling techniques and preprocessing steps to improve classification accuracy, especially when dealing with closely related classes.

### 2.6. Identification of Important Wavelengths (Without Mixed Bacteria)

The high rate of misclassification of SC1–SC7 samples as SC7 indicates significant spectral overlap among these categories, likely due to the dominance of SC7 characteristics in mixed bacterial samples. To investigate this phenomenon, five samples were randomly selected from the SC1–SC7 group. Infected regions were excised, prepared as specimen slides, and observed under a biological microscope (EX21, Sunny Optical Technology Co., Ltd., Ningbo, China) at 1000× magnification. Microscopic observations revealed that SC7 bacteria were significantly more abundant than SC1 bacteria in all the samples. This imbalance may be attributed to the rice variety’s greater resistance to SC1 or to experimental environmental conditions favoring SC7 proliferation. These factors likely contributed to the higher misclassification rate observed between SC7 and SC1–SC7 in the classification model. To address this issue and minimize interference from the mixed bacterial group, the SC1–SC7 group was removed, and the classification models were retrained using the remaining four datasets while maintaining the same preprocessing and feature extraction methods. The parameters of all modeling algorithms were optimized based on the five-class classification model configuration. For the 1DCNN model, the overall network structure remained unchanged, with modifications made only to the output layer to adapt to the transition from five to four categories.

Table 3 presents the classification performance of four models, namely PLSDA, KNN, RF, and 1DCNN, across the four categories: Health, Xoo, SC1, and SC7. After removing the SC1–SC7 group, all models showed improved performance. The 1DCNN model achieved F1 scores of 1.0000 for both the Health and SC1 groups, indicating perfect classification. This suggests that the spectral features of these two categories are sufficiently distinct to enable precise identification. While the classification of Xoo and SC7 remained challenging due to overlapping spectral features, the 1DCNN model consistently outperformed the other methods, attaining F1 scores of 0.9398 and 0.9412, respectively. These findings underscore the superior capability of deep learning approaches in distinguishing complex and closely related spectral data compared to traditional machine learning models, such as PLSDA, KNN, and RF.

Figure S3 presents the confusion matrix for the four-class classification model. After excluding the groups SC1–SC7, classification errors were primarily observed between Xoo and SC7, highlighting their high spectral similarity. Notably, all misclassifications in the 1DCNN model occurred within these two categories, underscoring the difficulty of distinguishing between Xoo and SC7. Overall, the 1DCNN model demonstrated the best overall performance in terms of class separation, achieving an accuracy of 97.06%, despite the challenges in distinguishing between Xoo and SC7. The PLSDA, KNN, and RF models exhibited lower classification accuracy compared to 1DCNN, with more frequent misclassifications and generally lower overall performance. These results highlight the superiority of 1DCNN in handling complex classification tasks involving high spectral similarity between classes.

## 3. Discussion

**Figure 6:**
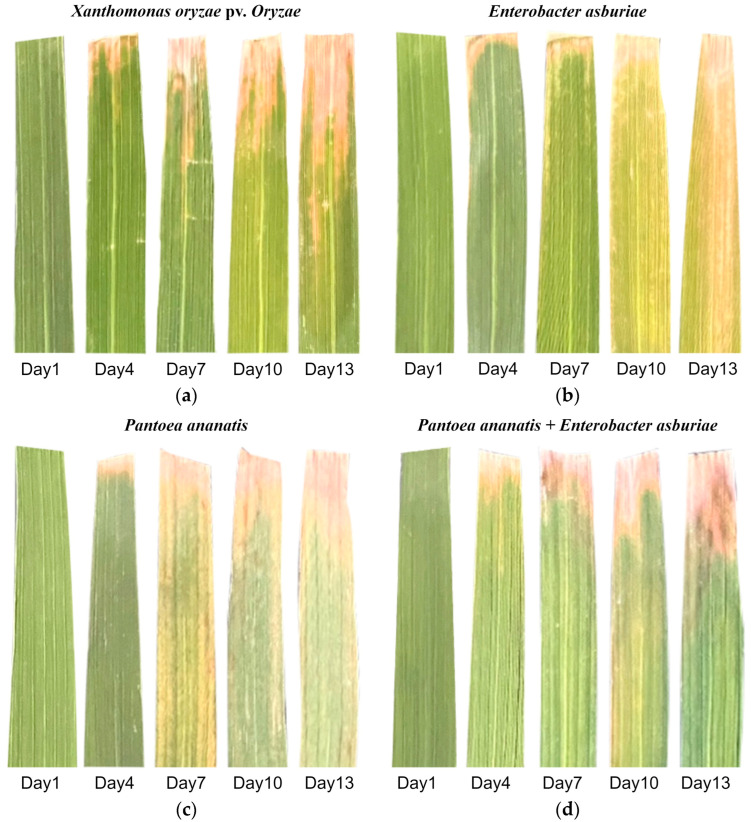
Disease progression in rice leaves infected with different pathogen strains: (**a**) *Xanthomonas oryzae* pv. *oryzae* (Xoo); (**b**) *Enterobacter asburiae* (SC1); (**c**) *Pantoea ananatis* (SC7); and (**d**) mixture of *Pantoea ananatis* and *Enterobacter asburiae* (SC1–SC7).

The present study demonstrates that HSI effectively distinguishes rice bacterial blight caused by different bacterial pathogens. Notably, the 1DCNN model outperformed traditional machine learning algorithms, including PLSDA, KNN, and RF, due to its advanced architecture, which allows for better feature extraction and higher generalization ability. This superior performance can be attributed to the model’s ability to capture more complex patterns in hyperspectral data compared to traditional methods. The results highlight that the advanced feature extraction capabilities of deep learning models enable them to outperform simpler models in terms of sensitivity and specificity. As shown in Figure 2, significant differences in reflectance between healthy and infected leaves were observed in specific spectral bands (notably 440–630 nm and 790–980 nm), although the overall spectral trends across all samples remain similar. This indicates that bacterial infection alters only a limited fraction of leaf components, leaving the intrinsic spectral properties largely intact. Despite the visual similarity of disease symptoms (Figure 6), SC1 exhibited distinct spectral characteristics compared to the other three infected groups, allowing for precise classification using machine learning models. Furthermore, when rice leaves were co-inoculated with SC1 and SC7, the spectral features were predominantly influenced by SC7, emphasizing the need to focus on differentiating between Xoo and SC7. Given the spectral and visual similarities between these two groups, as well as their comparable virulence levels [12], developing high-performance classification models remains critical for accurate discrimination.

This study prioritized achieving a balance between model efficiency and cost-effectiveness. In the five-class classification model (including SC1–SC7), the UVE-1DCNN combination achieved the best performance, with an accuracy of 86.11% and an F1 score of 0.8625. As shown in Figure S2, the primary source of misclassification occurred between SC1–SC7 and SC7. This misclassification can likely be attributed to the rice variety’s higher resistance to SC1 or to experimental conditions that favor the proliferation of SC7, resulting in SC7’s dominance within the SC1–SC7 samples. After removing SC1–SC7, the UVE-1DCNN model achieved an accuracy of 97.06% and an F1 score of 0.9703 on the four-class dataset. As illustrated in Figure S3, the remaining classification errors were mainly between SC7 and Xoo, which is a result consistent with previous studies [3,4,5,6,7,8,9,10,11,12]. These results highlight the need for better spectral feature separation, especially for closely related categories, and suggest that UVE-1DCNN is an effective choice for complex classification tasks, with its superior performance primarily driven by the model architecture’s ability to process hyperspectral data in a way that captures both spectral variance and intricate patterns. Although future optimizations in feature selection and model tuning may further improve performance, the 1DCNN model’s design offers significant advantages in terms of hyperspectral data processing and generalization ability, making it a promising approach for complex agricultural disease classification.

Additionally, this study explored the influence of five preprocessing techniques and three feature extraction methods on model performance. The results highlight the importance of selecting the most suitable preprocessing method for specific datasets and algorithms. For instance, in five-class full-spectrum modeling, MSC preprocessing improved PLSDA accuracy by 9.26% compared to raw data, demonstrating its strong compatibility with PLSDA. Conversely, SG preprocessing was more effective for KNN and RF models. Notably, 1DCNN achieved optimal performance without the need for preprocessing, underscoring its robustness in directly handling raw data. This distinct advantage sets 1DCNN apart from traditional models, which heavily rely on preprocessing to address noise sensitivity. Feature extraction methods further enhanced efficiency and reduced computational costs by identifying key wavelengths. Characteristic wavelength selection methods are vital for improving model performance by identifying the most relevant wavelengths for classification, thus reducing the dimensionality of the data and mitigating issues related to overfitting and computational burden. These techniques not only enhance the interpretability of the data but also enable more efficient model training. Dimensionality reduction can reveal hidden patterns in hyperspectral data that might be overlooked in high-dimensional spaces, ultimately improving classification performance and reducing computational costs. As shown in Figure 5, the critical bands selected by UVE, CARS, and SPA were primarily concentrated in the 420–490 nm, 610–670 nm, 780–850 nm, and 910–940 nm ranges. These findings align closely with previous research [27], which identified key spectral ranges for rice disease classification within 450–500 nm, 580–680 nm, and 720–940 nm.

While 1DCNN has achieved remarkable success, certain limitations should be acknowledged. A primary drawback of deep learning models is their reliance on large training datasets, which conflicts with the high cost of hyperspectral data acquisition. To address this challenge, GAN was utilized to generate synthetic one-dimensional spectral data, effectively expanding the dataset. PCA validated the high similarity between synthetic and real spectra, with minor deviations remaining within acceptable limits, consistent with previous findings in [30,31,32]. This approach not only mitigated overfitting but also enhanced classification accuracy, highlighting the potential of GAN-augmented data to improve the performance of 1DCNN in HSI-based plant disease detection. While this approach helps mitigate overfitting and enhances classification accuracy, care must be taken to ensure that synthetic data do not introduce bias or unrepresentative features. Future work should focus on optimizing GAN to generate more realistic synthetic data, incorporating additional factors such as environmental variability or disease progression, and exploring further data augmentation techniques to enhance model robustness and generalization ability.

However, this study has certain limitations. To minimize external variability, a single rice variety was selected, and artificial inoculation was conducted in a controlled indoor environment. While this enhanced experimental precision, it may have constrained the model’s generalizability and robustness under field conditions. Future research should explore diverse rice varieties and account for environmental variability to promote the practical application of this technology. Furthermore, increased data diversity demands more efficient and adaptive classification models, alongside techniques like cross-validation, to enhance accuracy, reliability, and robustness across diverse datasets. Additionally, SC7-induced rice bacterial blight is a relatively recent discovery, and its infection mechanisms and pathogenesis remain under investigation. This uncertainty may have introduced potential gaps in the current study, underscoring the importance of further research to refine and expand these findings.

## 4. Materials and Methods

### 4.1. Sample Preparation

The high-yielding, short-duration rice variety ‘Fanguisimiao’ was cultivated in an experimental field located in Guangdong Province, China (113°34′ E, 23°39′ N). At the 4–5 leaf stage, 20 pots of rice seedlings were transplanted from the field into pots for experimentation, with the soil sterilized at high temperatures prior to planting. Nitrogen fertilizer was applied in the field at the 2-leaf and 4-leaf stages to ensure sufficient nutrients during early growth. After transplanting, no additional fertilizer was applied, and the plants were watered regularly and quantitatively. For this study, *Pantoea ananatis*, *Enterobacter asburiae*, and *Xanthomonas oryzae* pv. *oryzae* were utilized as the causative agents of rice bacterial blight. All bacterial strains were provided by the Integrative Microbiology Research Centre, South China Agricultural University. Rice samples were divided into five groups and inoculated using a combination of leaf-cutting and spray methods. The groups included treatment with sterile water (Health), the *P. ananatis* strain SC7, *E. asburiae* strain SC1, *Xoo* strain PXO99A (Xoo), and a mixture of SC1 and SC7 (SC1–SC7).

As shown in Figure 6, the lesions on samples from the four groups inoculated with different pathogens gradually expanded over time. On the first day, no obvious lesions were observed in any of the samples. By the fourth day, small lesions began to appear in all groups. Lesion expansion in the SC7 and SC1–SC7 groups was notably faster than in the Xoo and SC1 groups. On the 7th day, lesion expansion in the SC7 and SC1–SC7 groups slowed, while on the 10th day, the lesion area in the Xoo and SC1 groups was similar to that in the SC7 and SC1–SC7 groups on the 7th day. After the 13th day, the rate of lesion expansion significantly decreased in all samples, and all lesions exhibited similar characteristics, such as leaf wilting and yellowing. Notably, in this experiment, wounds were created using the leaf-cutting method, followed by pathogen inoculation via spraying. The Xoo pathogen mainly entered through the wounds, whereas in the other three groups, in addition to lesion development from the wounds, the entire leaf gradually yellowed. This suggests that SC1 and SC7 might have a stronger ability to invade through the stomata. On the 14th day, 308 leaf samples (approximately 60 samples per group) were collected for analysis. Among the collected samples, although the lesions varied, it was difficult to distinguish the groups based on appearance alone.

### 4.2. HIS System and Spectra Acquisition

#### 4.2.1. HSI System

The HSI system comprised four key components: an imaging spectrometer, a light source, a transmission device, and dedicated data acquisition software. At its core was a spectrometer equipped with a CMOS area detector and lens (Specim FX10, Specim, Spectral Imaging Ltd., Oulu, Finland) operating within a wavelength range of 400–1000 nm. The spectrometer captured data across 224 wavelength points (average interval: 2.68 nm) with a spatial resolution of 1024 × 1024 pixels (pixel size: 8 × 8 μm) and a signal-to-noise ratio of 600:1. A 280W tungsten-halogen lamp (DECOSTAR 51S, Osram Corp., Munich, Germany) served as the light source, while a stepper motor (HXY-OFX01, Red Star Yang Technology Corp., China) ensured precise sample movement. The system was operated using Lumo-Scanner 4.8 software (Specim, Spectral Imaging Ltd., Oulu, Finland), which facilitated both data acquisition and analysis. To achieve optimal image clarity, key parameters were adjusted, including the conveyor belt speed (9.8 mm/s), CCD camera exposure time (2 ms), and the lens-to-sample distance (32.0 cm). The acquisition time for a single hyperspectral image was approximately 60 s, generating data across 224 spectral bands. After acquisition, raw spectral images were corrected to address underflow noise introduced during initial camera captures, ensuring quality and reliability.

#### 4.2.2. Spectra Extraction and Data Split

After collecting the hyperspectral data, black-and-white corrections were performed using a standard whiteboard and a black image via a calibration algorithm, thereby converting the data into reflectance mode [33]. The calibration process employed the following formula:

(1) $$R=\frac{R_{raw}-I_{B}}{I_{w}-I_{B}}$$

where *R* represents the corrected reflectance image, $R_{raw}$ is the raw reflectance image, $I_{W}$ is the white reference (intensity close to 1), and $I_{B}$ is the black reference (intensity close to 0).

**Figure 7:**
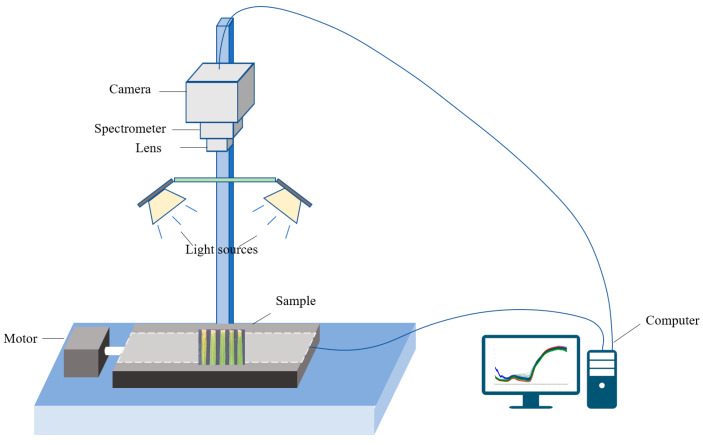
HSI system and data acquisition process.

After calibration, the region of interest (ROI) for each sample was selected, and the average spectral data were extracted, resulting in a spectral data matrix of size 308 × 224 (308 samples and 224 bands). All steps, including calibration, ROI selection, image segmentation, and spectral data extraction, were performed using ENVI 5.2 software. The process for obtaining hyperspectral data is illustrated in Figure 7.

### 4.3. Data Enhancement

A rich dataset is crucial for enabling models to learn the internal features of data effectively, thereby enhancing model stability and reducing the risk of overfitting. However, the limited number of spectral samples in this study falls short of the large dataset requirements typically needed to train deep learning models effectively. To address this limitation, a GAN, originally designed for generating synthetic images, was employed. The GAN framework comprises two key components: a Generator, which generates pseudo-spectral data, and a Discriminator, which evaluates its authenticity. Through this adversarial training process, the GAN learns the underlying feature distribution of the original data [32].

**Figure 8:**
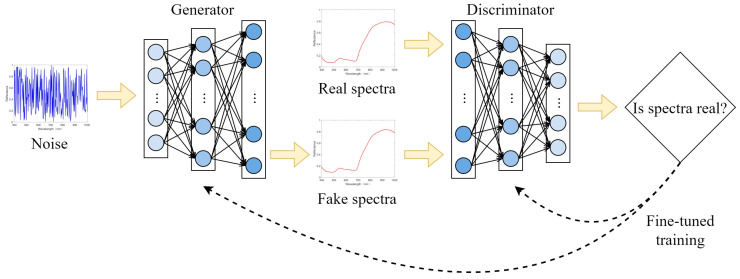
Architecture and training process of GAN.

In this study, as the spectra to be generated were one-dimensional and of relatively low complexity, a multi-layer neural network was adopted for the GAN architecture. The Generator was designed as a three-layer neural network with layer sizes of 128, 256, and 512, each utilizing a LeakyReLU activation function (alpha = 0.2). Similarly, the Discriminator consisted of layers with 512, 256, and 128, followed by a softmax layer, and employed a Sigmoid activation function. The model was trained using the Adam optimization algorithm with a batch size of 4, a learning rate of 0.00005, and a maximum of 2000 iterations. The GAN workflow is depicted in Figure 8.

To ensure the reliability and fairness of model evaluation, the testing set consisted solely of real samples. The real spectral dataset was randomly divided into training and testing sets in a 3:7 ratio, specifically consisting of 92 training samples and 216 test samples. To address data limitations and mitigate overfitting, a GAN was employed to augment the training set by generating 100 synthetic samples for each class, resulting in a total of 592 augmented training samples. The main objective of this study was to verify whether HSI combined with machine learning could identify similar symptomatic diseases caused by different pathogens. Since the accuracy requirement for the models was not very strict, and although hyperspectral data were difficult to acquire, their mathematical models are relatively simple, meaning that the augmented dataset in this study could be divided into training and testing sets at an approximately 7:3 ratio for analysis. The final data distribution is presented in Table 4.

### 4.4. Principal Component Analysis

PCA is a widely used technique for reducing the dimensionality of high-dimensional data. It transforms a set of potentially correlated variables into a new set of uncorrelated variables, called principal components, through orthogonal transformation [34]. Typically, the first two or three principal components capture the majority of the variance in the original data. We plotted the first principal component (PC-1) on the horizontal axis and the second (PC-2) on the vertical axis and included the third component (PC-3) on the depth axis so that the sample distribution could be visualized in two or three dimensions. This visualization aided in identifying key sample characteristics and clustering patterns, providing deeper insights into the underlying data structure.

### 4.5. Spectral Preprocessing

Preprocessing is a critical step in hyperspectral data analysis, designed to enhance data quality by reducing noise, correcting variations, and eliminating irrelevant features. In this study, the collected hyperspectral dataset exhibited challenges such as noise interference, baseline drift, intensity variability, and scattering effects, necessitating the evaluation of multiple preprocessing techniques. Commonly employed methods include the SG, NOR, BASE, SNV, and MSC [35]. SG smooths the data and reduces noise by fitting polynomials, effectively preserving critical signal features, such as peaks and valleys. NOR standardizes spectral intensity values, minimizing the variability caused by inconsistent light intensities or instrument drift. BASE removes signal drifts, enhancing signal-to-noise contrast and isolating the true spectral signal. SNV mitigates scattering effects and corrects sample-to-sample variability by normalizing each spectrum using its mean and standard deviation. Similarly, MSC compensates for multiplicative scattering effects, particularly in datasets with heterogeneous particle sizes or densities, improving the overall consistency of the spectral data. These preprocessing methods were systematically evaluated to determine the most effective approach for optimizing data quality and ensuring reliable downstream analysis. As shown in Figure S4, the spectral features of the augmented dataset changed to varying degrees after different preprocessing methods.

### 4.6. Characteristic Wavelengths Selection

To address the challenges of data correlation, redundancy, and covariance inherent in hyperspectral data, which stem from high dimensionality and large data volumes, three feature selection algorithms were employed: UVE, CARS, and SPA [36]. These methods aim to reduce model complexity while improving both classification accuracy and computational efficiency. UVE eliminates irrelevant variables by introducing random noise and comparing stability coefficients derived from partial least squares. Wavelengths with stability coefficients exceeding those of the noise are retained as key features. CARS employs a “survival of the fittest” strategy, iteratively discarding less informative wavelengths and retaining only those with the highest adaptability for model building. SPA addresses collinearity issues by iteratively selecting variables with the largest vector projections, thereby reducing dimensionality while preserving critical spectral information. Dimensionality reduction techniques further enhance this process by preserving the intrinsic data structure, which aids in revealing the most relevant features while reducing computational complexity [37]. By comparing these feature selection methods, this study identified the spectral bands most sensitive to classification models and optimized data dimensionality to enhance model performance.

### 4.7. Modeling Algorithm

The spectral information of rice leaves varies significantly under different bacterial stresses, necessitating the identification of key spectral bands from high-dimensional data and the development of accurate classification models. Drawing on prior studies [23,24,25,26,27], this study evaluated the performance of four classification approaches: PLSDA, KNN, RF, and 1DCNN.

PLSDA is a classification technique that integrates partial least squares regression with discriminant analysis. By projecting high-dimensional data into a lower-dimensional space, PLSDA maximizes the covariance between predictor variables and response classes, effectively addressing multicollinearity. This makes it particularly well suited for spectral data with numerous highly correlated variables [38]. As a result, PLSDA remains one of the most widely used methods in spectral analysis for achieving robust classification outcomes. KNN is a simple yet intuitive machine learning method applied to both classification and regression tasks. It predicts the label of a new sample by identifying its K nearest neighbors in the training dataset based on a defined distance metric, such as Euclidean distance. Despite its simplicity and ease of implementation, KNN can become computationally intensive with large datasets and is sensitive to noise, potentially impacting its classification accuracy. RF is a versatile ensemble learning method that performs exceptionally well in both classification and regression tasks. It constructs multiple decision trees during training and aggregates their predictions to enhance accuracy and control overfitting. Each tree is trained on a randomly selected subset of the data, allowing RF to handle large, high-dimensional datasets effectively while maintaining robustness against noise and overfitting.

**Figure 9:**
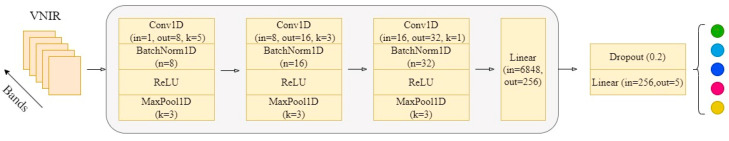
The 1DCNN model structure. Different categories are represented by different colors in the output.

1DCNN is a deep learning architecture optimized for sequential data, allowing the extraction of local patterns and temporal dependencies. This architecture performs convolutional operations along a single dimension, followed by pooling layers, to learn hierarchical features. Its ability to capture intricate relationships across spectral bands makes it particularly effective for analyzing hyperspectral data. The 1DCNN model architecture consists of three convolutional segments, each comprising a convolutional (Conv) layer (with kernel sizes: 5 × 1, 3 × 1, and 1 × 1, respectively) and a batch normalization (BN) layer, with channel counts of 8, 16, and 32. The extracted features were flattened and passed through a fully connected (FC) layer with 256 nodes. Cross-entropy was used as the loss function during the training phase. The Adam optimizer was employed for model training, with a learning rate set at 0.0001. The batch size and maximum number of training epochs were set to 64 and 600, respectively, while potential overfitting issues were addressed by setting the L2 weight decay parameter to 0.0001. The architecture of the developed 1DCNN model is illustrated in Figure 9.

### 4.8. Model Evaluation

To evaluate the reliability and robustness of the model, key performance metrics, including accuracy, precision, recall, and F1 score, were computed [39]. Accuracy, as defined in Equation (2), quantifies the proportion of correctly predicted instances and provides an overall measure of model effectiveness. Precision, as defined in Equation (3), represents the proportion of true positive predictions among all predicted positives, highlighting the model’s exactness in identifying positive cases. Recall, as defined in Equation (4), measures the proportion of actual positives correctly identified by the model, indicating its sensitivity. The F1 score, a harmonic mean of precision and recall defined in Equation (5), provides a balanced evaluation metric that is particularly useful for imbalanced datasets. Together, these metrics offer a comprehensive evaluation of the model’s performance, revealing its strengths and limitations.

(2) $$Accuracy=\frac{TP+TN}{TP+TN+FP+FN}$$

(3) $$Precision=\frac{TP}{TP+FP}$$

(4) $$Recall=\frac{TP}{TP+FN}$$

(5) $$F1=\frac{2\times Precision\times Recall}{Precision+Recall}$$

where *TP* (true positive) represents correctly predicted positive cases, *TN* (true negative) denotes correctly predicted negative cases, *FP* (false positive) refers to incorrectly predicted positives, and *FN* (false negative) corresponds to incorrectly predicted negatives.

### 4.9. Computational Environment

Spectral preprocessing and characteristic wavelength selection were performed using The Unscrambler X 10.4 and MATLAB 2021a, respectively. All models were implemented in a Windows 10 environment using Python 3.9, PyTorch 1.10, and scikit-learn libraries. The computational setup comprised an Intel Xeon Gold 6142 CPU and an NVIDIA RTX 3080 GPU with 10.5 GB of memory, ensuring sufficient computational capacity for high-dimensional spectral data analysis.

## 5. Conclusions

This study integrated HSI and GAN to construct a spectral dataset comprising rice leaves inoculated with *Xoo*, *P. ananatis*, *E. asburiae*, a mixed pathogen group (*P. ananatis* + *E. asburiae*), as well as healthy samples, to support modeling requirements. A UVE-1DCNN model was developed to classify rice leaves based on various pathogen infections, with its superior performance validated through comparisons across various preprocessing techniques, feature extraction methods, and machine learning algorithms. In samples inoculated with mixed bacterial pathogens, both lesion characteristics and spectral features closely resembled those of the *P. ananatis*-infected samples. This phenomenon may be attributed to the rice variety’s resistance to *E. asburiae* or environmental conditions favoring the proliferation of *P. ananatis*, which became dominant within the mixed-infection samples. Upon removing the mixed-infection group, the four-class classification model exhibited a notable improvement in accuracy, reaching 97.06%. The model achieved the precise classification of all four categories, effectively distinguishing healthy leaves as well as those infected by *P. ananatis*, *E. asburiae*, and *Xoo*. A comparative analysis of feature selection methods identified critical wavelength ranges (420–490 nm, 610–670 nm, 780–850 nm, and 910–940 nm) as essential for differentiating this emerging bacterial blight. This study proposes a precise, non-destructive, and rapid approach for identifying rice bacterial blight symptoms caused by various bacterial pathogens, including *Xanthomonas oryzae* pv. *oryzae*, *P. ananatis,* and *E. asburiae,* offering valuable insights into its pathogenic mechanisms and supporting the development of effective disease control strategies.

## Figures and Tables

**Table 1:** Comparison of different combinations of feature extraction and modeling methods.

Algorithm	Parameter	Preprocessing Methods	Identification Accuracy	
			Training Set	Testing Set
PLSDA	PCs = 10	UVE	0.8530	0.7824
	PCs = 10	CARS	0.8581	0.7731
	PCs = 4	SPA	0.8429	0.7500
KNN	k = 2	UVE	0.8125	0.7824
	k = 2	CARS	0.8192	0.7824
	k = 2	SPA	0.8378	0.7917
RF	n = 50, depth = 5	UVE	0.8125	0.7361
	n = 50, depth = 6	CARS	0.8057	0.7454
	n = 50, depth = 5	SPA	0.8311	0.7639
1DCNN	/	UVE	0.9088	0.8611
		CARS	0.8902	0.8287
		SPA	0.9037	0.8472

**Table 2:** Comparison of different modeling methods with optimal parameters for the identification of five types of diseases.

Label	Index	PLSDA	KNN	RF	1DCNN
Health	Precision	1.0000	1.0000	1.0000	1.0000
	Recall	1.0000	1.0000	1.0000	1.0000
	F1	1.0000	1.0000	1.0000	1.0000
Xoo	Precision	0.7250	0.7234	0.7209	0.8409
	Recall	0.7073	0.8293	0.7561	0.9024
	F1	0.7160	0.7727	0.7381	0.8706
SC1	Precision	0.9730	0.8889	0.8409	1.0000
	Recall	0.8000	0.8333	0.8222	0.9556
	F1	0.8780	0.8602	0.8315	0.9773
SC7	Precision	0.6458	0.6170	0.6275	0.7509
	Recall	0.7209	0.6744	0.7442	0.8372
	F1	0.6813	0.6444	0.6809	0.7660
SC1–SC7	Precision	0.6400	0.6923	0.6486	0.7838
	Recall	0.6956	0.5870	0.5217	0.6304
	F1	0.6667	0.6353	0.5783	0.6988

**Table 3:** Comparison of different modeling methods with optimal parameters for identifying four groups of samples.

Label	Index	PLSDA	KNN	RF	1DCNN
Health	Precision	1.0000	1.0000	1.0000	1.0000
	Recall	1.0000	1.0000	1.0000	1.0000
	F1	1.0000	1.0000	1.0000	1.0000
Xoo	Precision	0.9000	0.7917	0.8605	0.9286
	Recall	0.8780	0.9268	0.9024	0.9512
	F1	0.8889	0.8539	0.8810	0.9398
SC1	Precision	0.9773	0.9524	1.0000	1.0000
	Recall	0.9556	0.8889	0.9111	1.0000
	F1	0.9663	0.9195	0.9535	1.0000
SC7	Precision	0.8667	0.8974	0.8444	0.9524
	Recall	0.9070	0.8140	0.8837	0.9302
	F1	0.8864	0.8537	0.8636	0.9412

**Table 4:** Details of rice diseases in the dataset with samples from each class.

Label	Raw Data	Training Set		Testing Set
		Raw Data	Enhanced Data	
Health	58	17	117	41
Xoo	58	17	117	41
SC1	64	19	119	45
SC7	62	19	119	43
SC1–SC7	66	20	120	46
Total	308	92	592	216

## Data Availability

The data presented in this study are available upon request from the corresponding author. Data available on request due to privacy.

## Supplementary Materials

The following supporting information can be downloaded at https://www.mdpi.com/article/10.3390/plants14050733/s1. Table S1: Comparison of different preprocessing and modeling methods based on full-spectrum information. RAW represents raw spectral data. Figure S1: Generated spectra under different iterations and real spectrum: (a) epoch = 100; (b) epoch = 400; (c) epoch = 800; (d) epoch = 1200; (e) epoch = 2000; and (f) real spectrum. Figure S2: Confusion matrices of different modeling methods with optimal parameters for the identification of five groups of samples: (a) PLSDA; (b) KNN; (c) RF; and (d) 1DCNN. Figure S3: Confusion matrix of different modeling methods with optimal parameters for the identification of four groups of samples: (a) PLSDA; (b) KNN; (c) RF; and (d) 1DCNN. Figure S4: Spectral characteristic curves after different preprocessing methods: (a) RAW; (b) SG; (c) NOR; (d) BASE; (e) SNV; and (f) MSC.

## Abbreviations

The following abbreviations are used in this manuscript:

*Xoo*	*Xanthomonas oryzae* pv. *oryzae*
*P. ananatis*	*Pantoea ananatis*
*E. asburiae*	*Enterobacter asburiae*
PCR	Polymerase chain reaction
HSI	Hyperspectral imaging
PLSDA	Partial least squares discriminant analysis
PCA	Principal component analysis
KNN	K-nearest neighbors
RF	Random forest
1DCNN	One-dimensional convolutional neural networks
GAN	Generative Adversarial Networks
SG	Savitzky–Golay filter
NOR	Normalization
BASE	Baseline correction
SNV	Standard normal variate
MSC	Multiplicative scatter correction
UVE	Uninformative variable elimination
CARS	Competitive adaptive reweighted sampling
SPA	Successive projections algorithm
ROI	Region of interest
Conv	Convolutional
BN	Batch normalization
FC	Fully connected
*TP*	True positive
*TN*	True negative
*FP*	False positive
*FN*	False negative
