# Supplementary material for: Hyperspectral Imaging and Machine Learning for Diagnosing Rice Bacterial Blight Symptoms Caused by Xanthomonas oryzae pv. oryzae, Pantoea ananatis and Enterobacter asburiae
Source: Plants (Basel). 2025 Feb 27;14(5):733. doi: 10.3390/plants14050733 (PMC11901819; doi:10.3390/plants14050733)
Supplement: Supplementary file 1 [file plants-14-00733-s001.zip › plants-3494791-supplementary.pdf]

Supplementary material

Table S1. Comparison of different preprocessing and modeling methods based on full spectrum information. RAW represents raw spectral data.

Algorithm	Parameter	Preprocessing Methods	Identification Accuracy	
			Training Set	Testing Set
PLSDA	PCs = 9	Raw	0.7517	0.7176
	PCs = 9	SG	0.8108	0.7778
	PCs = 9	NOR	0.7770	0.7315
	PCs = 8	BASE	0.7753	0.7222
	PCs = 9	SNV	0.8108	0.7731
	PCs = 9	MSC	0.8530	**0.8102**
KNN	k = 2	Raw	0.7703	0.7315
	k = 2	SG	0.8429	**0.8102**
	k = 3	NOR	0.8125	0.7778
	k = 2	BASE	0.8125	0.7778
	k = 3	SNV	0.8193	0.7454
	k = 3	MSC	0.8429	0.7731
RF	n = 50, depth = 4	Raw	0.7517	0.6991
	n = 70, depth = 5	SG	0.8446	**0.7917**
	n = 55, depth = 4	NOR	0.8260	0.7500
	n = 65, depth = 4	BASE	0.8074	0.7269
	n = 65, depth = 4	SNV	0.7956	0.7269
	n = 70, depth = 5	MSC	0.8074	0.7546
1DCNN	/	Raw	0.9206	**0.8889**
		SG	0.9105	0.8519
		NOR	0.8986	0.8611
		BASE	0.9020	0.8565
		SNV	0.9105	0.8519
		MSC	0.8902	0.8380

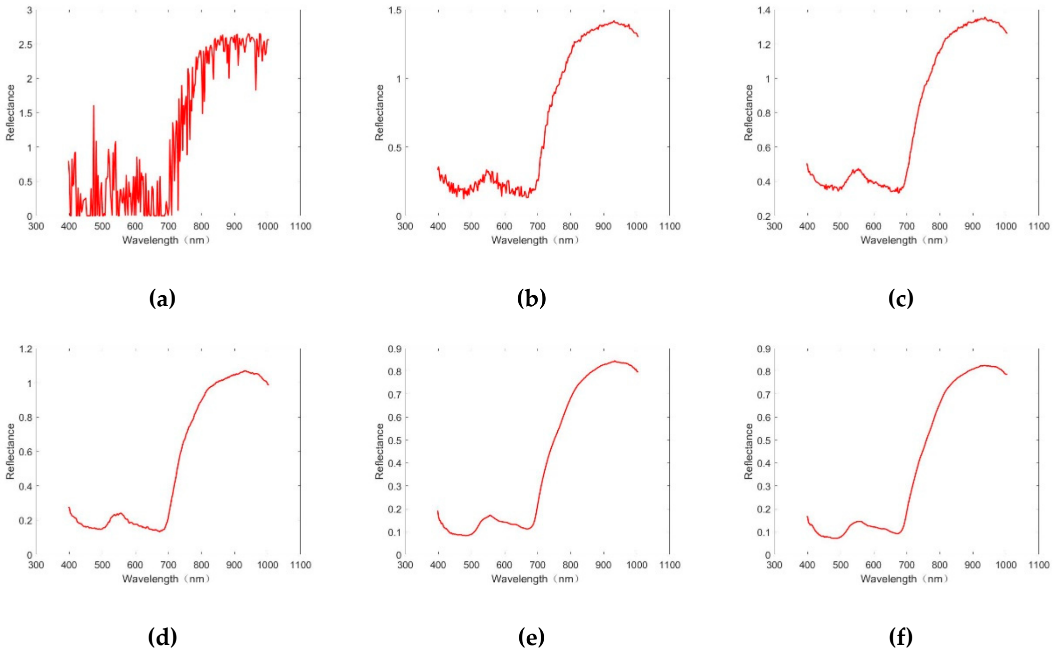

Figure S1. Generated spectra under different iterations and real spectrum: (**a**) epoch = 100; (**b**) epoch = 400; (**c**) epoch = 800; (**d**) epoch = 1200; (**e**) epoch = 2000 and (**f**) real spectrum.

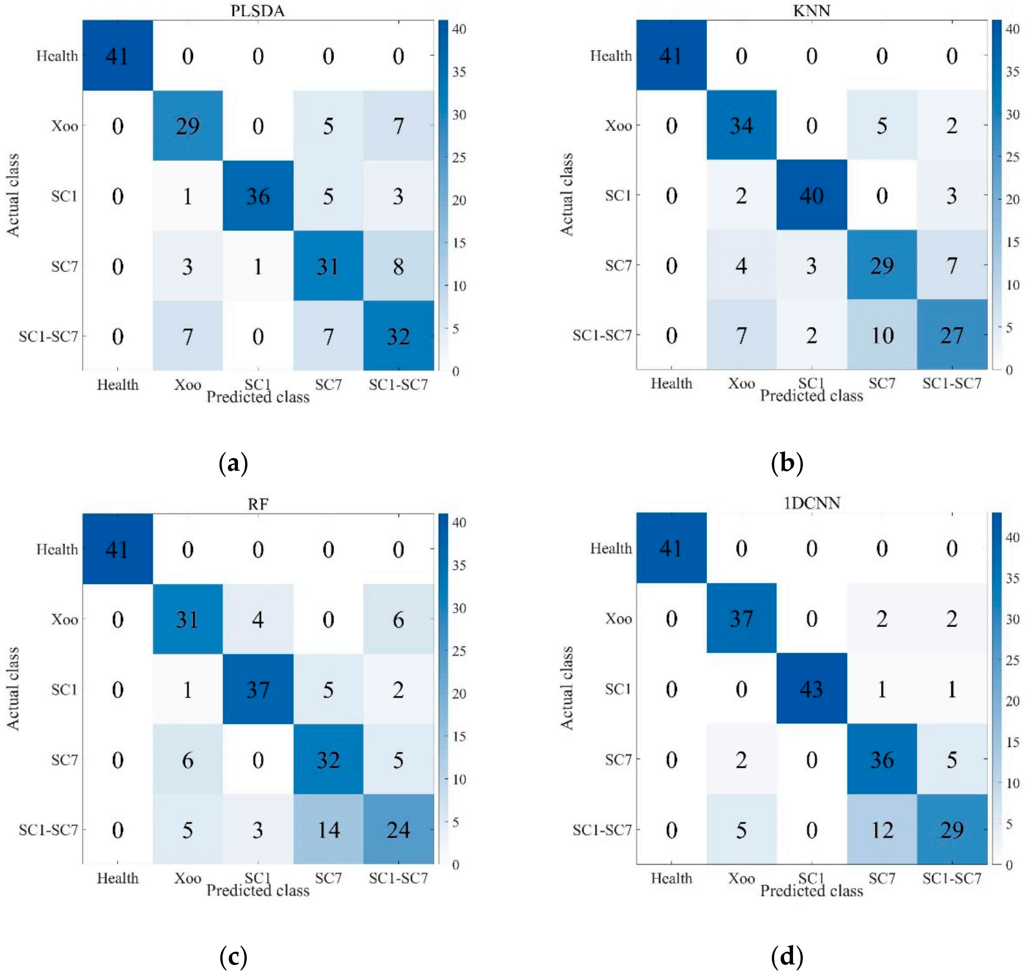

Figure S2. Confusion matrices of different modeling methods with optimal parameters for identifying five groups of samples: (**a**) PLSDA; (**b**) KNN; (**c**) RF and (**d**) 1DCNN.

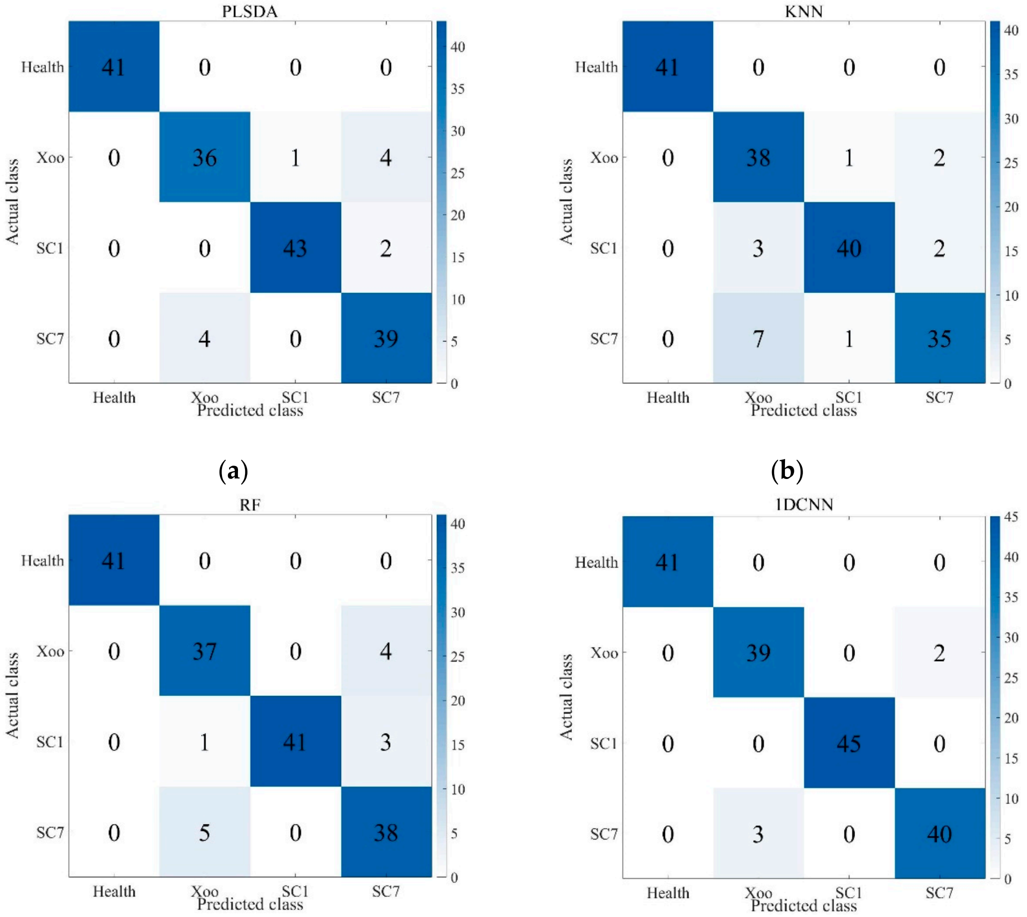

(c) (d)

Figure S3. Confusion matrix of different modeling methods with optimal parameters for identifying four groups of samples: (**a**) PLSDA; (**b**) KNN; (**c**) RF and (**d**) 1DCNN.

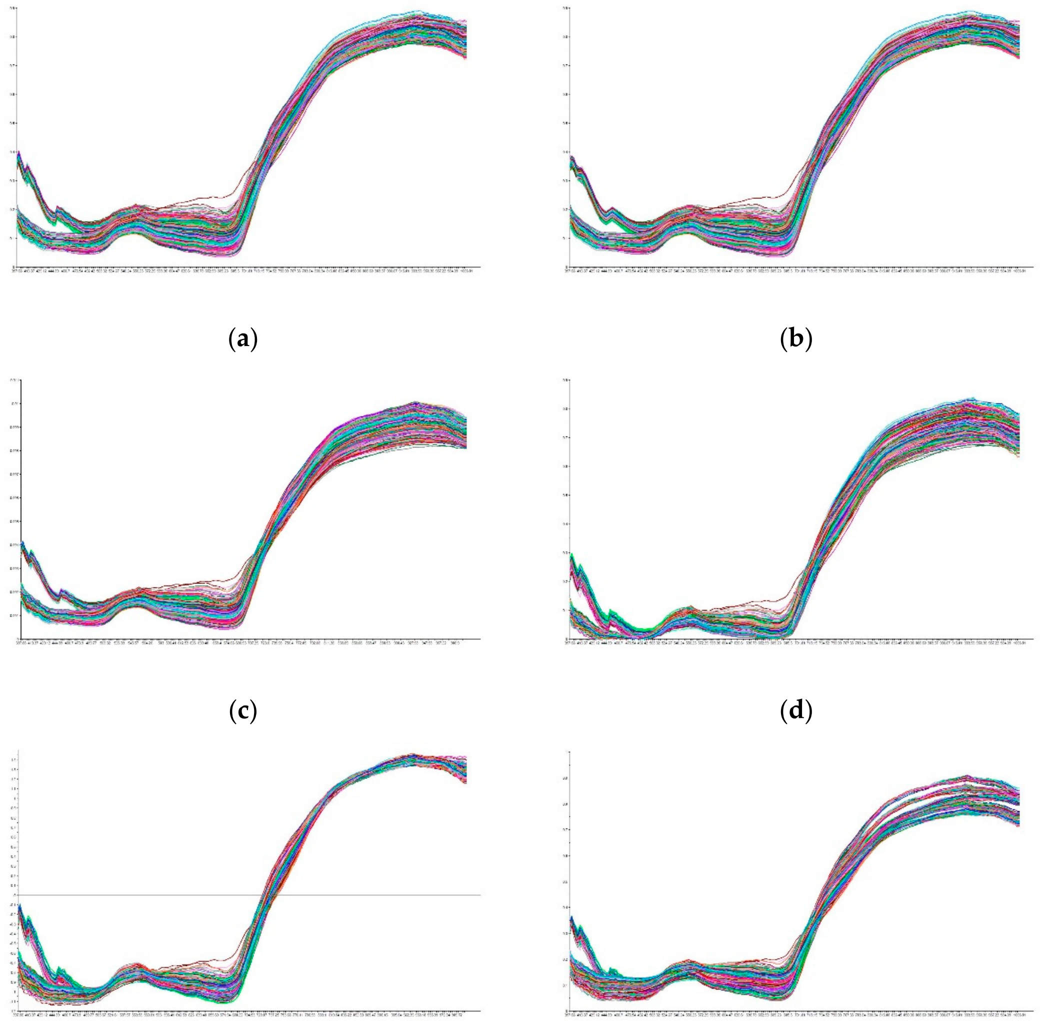

(**a**) (**b**)

(**c**) (**d**)

(**e**) (**f**)

Figure S4. Spectral characteristic curves after different preprocessing methods: (**a**) RAW; (**b**) SG; (**c**) NOR; (**d**) BASE; (**e**) SNV and (**f**) MSC.
